# Comparison of the Cepheid Xpert HPV test and the HC2 High-Risk HPV DNA Test for detection of high-risk HPV infection in cervical smear samples in SurePath preservative fluid

**DOI:** 10.1099/jmm.0.000723

**Published:** 2018-03-26

**Authors:** Ali A. Rabaan, Shatha A. Alfaraj, Mohammed A. Alkhalifah

**Affiliations:** ^1^​ Molecular Diagnostic Laboratory, Johns Hopkins Aramco Healthcare, Dhahran 31311, Saudi Arabia; ^2^​ Histopathology Laboratory, Laboratory and Blood Bank Department, Qatif Central Hospital, Qatif 31911, Saudi Arabia; ^3^​ Laboratory and Blood Bank Department, Qatif Central Hospital, Qatif 31911, Saudi Arabia

**Keywords:** Human papillomavirus, cervical cancer, Cepheid Xpert HPV test, Saudi Arabia

## Abstract

**Purpose:**

Cytological and histological cervical screening methods for human papillomavirus may be subjective. Current guidelines recommend the use of direct human papillomavirus screening by molecular methods in conjunction with cytology for the detection of high-risk human papillomavirus types with carcinogenic potential. In this study, we compared the performance of the molecular Cepheid Xpert HPV test to the FDA-approved HC2 High-Risk HPV DNA Test on samples from patients presenting for cervical screening, regardless of the cytology results, in which cervical cell samples were originally collected for Papanicolaou (Pap) smear specimens in Becton Dickinson (BD) SurePath preservative fluid.

**Methodology:**

Cervical cells were obtained for Pap smear specimens from 343 women attending Qatif Central Hospital in Saudi Arabia for cervical cancer screening using a Cytobrush Plus GT and immersed in BD SurePath preservative fluid in BD SurePath collection vials. The study was carried out between December 2015 and July 2016.

**Results:**

The Xpert HPV test was positive in 27 (7.9 %) of the samples. The HC2 High-Risk HPV DNA Test was positive in 32 (9.3 %) of the samples. The most common HPV types according to the Xpert HPV test were HPV other types, either alone (*n*=15) or in combination with HPV16 (*n*=3). The overall concordance rate between the tests was 98.5 %. The positive concordance was 84.4 %.

**Conclusion:**

The Xpert HPV test is convenient to use on cervical cell samples collected for Pap smear specimens in BD SurePath preservative fluid within an hour and is a viable alternative to the HC2 High-Risk HPV DNA Test for HPV testing.

## Introduction

Human papillomavirus (HPV) is the broad term for a group of approximately 120 related strains or types of papillomaviruses that can infect humans [1–3]. A number of high-risk types can cause precancerous lesions and are responsible foralmost all cases of cervical cancer, most cases of cancer of the anus and oropharynx, and many cases of penile, vulvar and vaginal cancer [1, 2]. Approximately 75 to 80 % of sexually active individuals are likely to suffer a HPV infection in their lifetime [4, 5]. The HPV types linked to cancer all belong to the family *α*-*Papillomaviridae* [6].

The International Agency for Research on Cancer (IARC) divides HPV types into groups according to their carcinogenic potential [1, 6, 7]. Group 1 contains 12 types defined as being carcinogenic to humans, including HPV16, HPV18, HPV31, HPV33, HPV35, HPV39, HPV45, HPV51, HPV52, HPV56, HPV58 and HPV59 [1, 6]. Another type, HPV68 in group 2A, is defined as probably carcinogenic [1, 6]. These 13 high-risk HPV (hrHPV) types have been linked to 96 % of cervical cancers, with HPV16 and HPV18 together being responsible for approximately 70 % of cases globally. A further 2.6 % of cases are probably attributable to 12 members of group 2B, including HPV66, which are defined as probable carcinogens [1, 6]. The risk factors for women for HPV infection include early age of onset of sexual activity, more than one sexual partner in the preceding year, or coinfection with herpes simplex, *Chlamydia trachomatis* or bacterial vaginosis [1, 8]. Women aged under 25 years have the highest incidence of HPV infection, with another peak between 35–54 years [1]. Incidence declines thereafter [1].

Regular cervical cancer screening is important for early detection and treatment. Currently, the most common screening method involves the collection of cervical cells for staining with Papanicolaou (Pap) stain and microscopic examination [1]. This can detect atypical squamous cells (ASC), which can be precancerous, including ASC of undetermined significance (ASC-US) and ASC-H, which have higher precancerous risk potential than ASC-US, as well as low-grade squamous intraepithelial lesions (LSIL), or high-grade intraepithelial lesions (HSIL) [1, 9]. Regular cytology screening programmes based on Pap staining have been successful in many developed countries for the early detection of precancerous lesions and cervical cancer. For abnormal findings, colposcopy and biopsy samples will be taken and histologically examined and defined according to the LAST 2012 criteria [9].

However, there are some concerns that the cytological and histological screening methods may be subjective and that there is considerable variation in interpretation between laboratories [1]. Thus the current guidelines recommend the use of direct HPV screening by molecular methods to detect the presence of HPV, in conjunction with cytology, with different recommendations depending on age group and degree of risk [1, 10, 11]. As direct HPV testing is now thought to detect more high-grade precancerous lesions than classic Pap smears, molecular HPV tests are being adopted as the primary screening method in some places [12, 13]. Many different HPV molecular tests have been approved by the United States (US) Food and Drug Administration (FDA) and/or clinically validated by the Meijer criteria for use in Europe or Canada [12, 13]. Most give a pooled result, indicating the presence or absence of group 1 HPV types, sometimes with the addition of HPV68 and/or HPV66. One approved molecular test is the Hybrid Capture 2 (HC2) High-Risk HPV DNA Test, developed by Digene and currently marketed by Qiagen; it is often used as the comparator test for proposed new diagnostic HPV detection tests [1] It generates a pooled result based on the expression of the 12 group 1 HPV types and HPV68 [1]. The Cepheid Xpert HPV test is not yet FDA-approved, but can detect the 12 Group 1 HPV types, as well as HPV68 and HPV66, within an hour, as opposed to the currently approved batch tests, which take several hours to complete [14]. It can distinguish between the different HPV types present rather than giving a pooled result. Previous studies suggested that the Cepheid Xpert HPV test gives similar results to the FDA-approved Cobas 4800 test, which is based on multiplex PCR and nucleic acid hybridization [14]. Our previous study on 168 ASC-US samples from a Saudi Arabian hospital showed that the Cepheid Xpert HPV test had 98.2 % overall concordance with the HC2 High-Risk HPV DNA Test, and 91 % positive concordance [15].

In this study we compared the performance of the Cepheid Xpert HPV test to the HC2 High-Risk HPV DNA Test using samples from all patients presenting for cervical screening, in which cervical cell samples were originally collected for Pap smear specimens in Becton Dickinson (BD) SurePath preservative fluid. This is the first time, to the best of our knowledge, that these tests have been performed directly on cell samples routinely collected for a standard Pap stain in this reagent.

## Methods

### Study population and design

The study population included all women attending Qatif Central Hospital for cervical cancer screening, regardless of the cytology results. The study was approved by Qatif Central Hospital Institutional Review Board (IRB). Samples were taken from 343 women, of whom 338 (98.5 %) were Saudi nationals. The mean age±standard deviation (sd) was 40.6±10.7 years and the age range was 20–80 years. The distribution by age group was 20–29 years, *n*=54; 30–39 years, *n*=109; 40–49 years, *n*=115; 50–59 years, *n*=53; ≥60 years, *n*=12.

Cervical cells were obtained for Pap smear specimens using a Cytobrush Plus GT and immersed in BD SurePath preservative fluid in BD SurePath collection vials. The study was carried out between December 2015 and July 2016.

### HC2 High-Risk HPV DNA Test

For the HC2 High-Risk HPV DNA Test, samples were used directly from the SurePath collection vials for HC2 High-Risk HPV DNA Test analysis. Four millilitres of sample were processed directly using the Digene HC2 Sample Conversion kit (Qiagen) and the HC2 High-Risk HPV DNA Test was carried out according to the manufacturer’s instructions, as in Rabaan *et al.* [15].

### Cepheid Xpert HPV test

Cell solution taken directly from the SurePath collection vials was homegenized and transferred to BD centrifuge tubes (c-tubes) containing pre-aliquoted density reagent, using the BD PrepMate Automated Accessory, according to the manufacturer’s instructions (Becton Dickinson). Two different centrifugation steps were then carried out to produce a concentrated and enriched cell pellet, as normally used for SlidePrep and cytological screening. In this study, 1 ml of enriched pellet suspension was placed in an Xpert HPV cartridge (Cepheid, Sunnyvale, CA, USA) and run and analysed on the GeneXpert Dx system for second-generation real-time PCR analysis, according to the manufacturer’s instructions, as in Rabaan *et al.* [15].

### Statistical tests

We calculated the overall concordance between the HC2 High-Risk HPV DNA Test and the Cepheid Xpert HPV test, and the positive concordance between the tests. The McNemar chi-squared test was applied to test the statistical significance of the discordance between the tests; *P*<0.05 was accepted as significant. We also calculated the percentage agreement between the two methods using the Kappa statistic. The results were interpreted based on the guidelines that negative Cohen’s kappa means no agreement between methods, between 0 and 0.20 indicates slight agreement, 0.21–0.40 indicates fair agreement, 0.41–0.60 indicates moderate agreement, 0.61–0.80 indicates substantial agreement and 0.81–1 indicates almost perfect agreement [16, 17]. The proportion of HPV-positive versus HPV-negative results by age group was compared by chi-squared analysis.

## Results

A total of 343 samples were tested from women undergoing cervical cancer screening, regardless of the cytology results. The results of both screening tests are summarised in Table 1. The Cepheid Xpert HPV test was positive in 27 (7.9 %) of the samples. The HC2 High-Risk HPV DNA Test was positive in 32 (9.3 %) of the samples. The most common HPV types according to the Cepheid Xpert HPV test were HPV other types, either alone (*n*=15) or in combination with HPV16 (*n*=3) (Table 1).

**Table 1.** Results of the HC2 High-Risk HPV DNA Test and the Cepheid Xpert HPV test

**Test**	**HPV type**	**Number (total=343) (percentage)**
HC2 High-Risk HPV DNA Test	Positive (high-risk HPV)	32 (9.3 %)
		
Cepheid Xpert HPV test	HPV16	5 (1.5 %)
	HPV18/45	4 (1.2 %)
	HPV16 and others (P3)	1 (0.3 %)
	HPV16 and others (P4)	0
	HPV16 and others (P5)	2 (0.6 %)
	HPV others (P3)	6 (1.8 %)
	HPV others (P4)	3 (0.9 %)
	HPV others (P5)	6 (1.8 %)
	Total (Cepheid)	27

Table 2 shows the overall concordance rate between the HC2 High-Risk HPV DNA Test and the Cepheid Xpert HPV test. The overall concordance was 98.5 %. The McNemar chi-square statistic was 3.2 and the *P* value was 0.07, indicating that there was no significant degree of discordance. Cohen’s kappa was 0.907 (95 % CI, 0.827–0.988), indicating almost perfect agreement. The positive concordance was 84.4 %.

**Table 2.** Concordance between the results for the HC2 High-Risk HPV DNA Test and the Cepheid Xpert HPV test

	**HC2 High-Risk HPV**		
**Cepheid Xpert HPV**	**Positive**	**Negative**	**Total**
Positive	27	0	**27**
Negative	5	311	**316**
Total	**32**	**311**	**343**

McNemar chi-square statistic, 3.2; *P* value (two-tail), 0.07.

When the 338 samples for which the two tests gave the same result were considered by age group (20–29 years, 30–39 years, 40–49 years, 50–59 years and ≥60 years), there was no significant difference in the proportion of positive versus negative results (chi-square statistic=2.3275, *P*=0.678).

There were a range of clinical signs for the 27 samples that were positive for both tests, including irregular menstruation (*n*=6), vaginitis (*n*=6), post-coital bleeding (*n*=3), uterine fibroid/polyp (*n*=3), menorrhagia (*n*=2), vaginal discharge (*n*=2), abdominal pain (*n*=1), Bartholin cyst (*n*=1) and infertility (*n*=1), in addition to attendance for routine screening (*n*=2). For 26 of these 27 samples, pathology results were available. For 21 samples there was nil pathology, for 1 nil with atrophy and for 1 nil with severe inflammation. Three samples were identified as ASC-H (all HPV16-positive), and two were identified by histology as carcinomas. The pathology was nil for the five cases that were positive by HC2 but negative by Cepheid.

## Discussion

As in our previous study, we observed high concordance and reasonable positive concordance between the HC2 High-Risk HPV DNA Test and the Cepheid Xpert HPV test [15]. In that study on ASC-US samples, the Cepheid Xpert HPV test was positive in 17.8 % of samples, as compared to 7.9 % in this study, while the HC2 High-Risk HPV DNA Test was positive in 19.6 % of samples, as compared to 9.3 % in this study. The current study, however, differed in that all cervical cell samples from women with a wide range of clinical signs presenting for cervical screening were included, regardless of cytology, whereas the previous study was confined to ASC-US samples. Thus this study is likely to have given a closer estimation of overall hrHPV levels in women in Saudi Arabia.

At present, there is little information on the prevalence and type of distribution of HPV in Saudi Arabia. Cervical cancer levels are low compared to many other countries, but women who present with cervical cancer are often at an advanced stage in which they require intensive chemotherapy and radiation treatment, due to the lack of a national screening programme [17–20]. Previous studies have suggested an overall hrHPV prevalence of 5.6 % in the western region of Saudi Arabia, as measured by the HC2 High-Risk HPV DNA Test [20]. Thus the levels of hrHPV infection among women in Saudi Arabia, like the levels of cervical cancer, appear to be lower than in countries such as the United States, and comparable to those in other Arab states. There was no apparent association of positive results with any particular age group in our study. The most prevalent hrHPV types were ‘HPV other’, which encompasses P3 (primary for the pooled results of HPV types 31, 33, 35 52 or 58), P4 (primary for the pooled results of HPV type 51 or 59), or P5 (primary for the pooled results of HPV types 39, 56, 66 or 68). This is consistent with previous observations [15]. Many of these types have been shown to be more prevalent in HSIL versus cervical cancer [21]

In previous studies on Saudi women with cervical cancer, there was hrHPV infection in 89.0–95.5 % of cases. HPV16 was by far the most prevalent, followed by HPV18, HPV45 and HPV-33 [22]. The prevalence of HPV-16 and HPV-18 in cervical cancer cases suggests that an effective screening and/or vaccination programme in Saudi Arabia would reduce the prevalence of cervical cancer and allow detection and treatment at an earlier stage. Our results indicated a higher prevalence of ‘other’ hrHPV types, although HPV16 and HPV18 infections were also detected. Two samples that were positive by both tests and identified as carcinomas by histology were HPV16-positive.

In this study we used cervical cell samples that were originally collected for Pap smear specimens in Becton Dickinson (BD) SurePath preservative fluid. The overall concordance between the tests, and a comparison with the prevalence results from other studies, suggests that this did not interfere with the tests and that they worked efficiently under these conditions [15, 20]. This would be a convenient and practical way of using the Cepheid Xpert HPV test in conjunction with conventional Pap screening, as the same samples could be used for both tests.

Although the HC2 High-Risk HPV DNA Test was positive in five samples that gave a negative result with the Cepheid Xpert HPV test, it has been previously noted that the HC2 High-Risk HPV DNA Test has a false positive rate of 5 % in tests where PCR does not detect HPV DNA [23–25]. The probe technology used in the HC2 High-Risk HPV DNA Test is also subject to cross-reactivity with non-targeted HPV types, including both non-carcinogenic types as well as possibly carcinogenic types [23]. Nevertheless, the discordance in the results for these five samples represents a limitation to our study that requires further follow-up to characterize these cases by alternative methods. The pathology was nil for all five of these discordant cases. The determination of viral load in discordant cases could give an insight into whether there is a lower viral load than in the concordant cases. In studies using the validation of human papillomavirus (HPV) genotyping tests (VALGENT) study framework, which is designed to allow test comparison and the validation of HPV assays, the Cepheid Xpert HPV test was validated for screening, as it showed comparable accuracy for cervical pre-cancer as compared to standard comparator tests, i.e. HC2 and GP5+/6+-EIA [26, 27].

The Cepheid Xpert HPV test has the advantage of providing information on the presence of individual HPV types rather than a pooled result. It is also more rapid to perform, giving results within an hour as compared to taking several hours, as with the HC2 High-Risk HPV DNA Test, meaning that the patient can be referred for colposcopy immediately. Other studies have also indicated substantial agreement between the HC2 High-Risk HPV DNA Test test and the Cepheid Xpert HPV test. For example, in a recent assessment of anal hrHPV prevalence in 199 HIV-positive women in South Africa there was 86.7 % agreement between the tests [28], while in a colposcopy referral population of 708 women in the United States the Cepheid Xpert HPV test was shown to be sensitive and reliable compared to both the HC2 High-Risk HPV DNA Test test and the FDA-approved Cobas HPV test (Roche Molecular Systems) [29]. Another advantage of the Cepheid Xpert HPV test is its recently demonstrated utility in testing formalin-fixed paraffin-embedded (FFPE) samples [30, 31].

In conclusion, in this study we have shown that the Cepheid Xpert HPV test can be used on cervical cell samples collected for Pap smear specimens in BD SurePath preservative fluid and shows good agreement with the FDA-approved HC2 High-Risk HPV DNA Test test. Thus the Cepheid Xpert HPV test is a viable alternative to the HC2 High-Risk HPV DNA Test test and can be conveniently performed on samples taken routinely for Pap smear to generate a result within an hour.
